# Defining functional spatial boundaries using a spatial release from masking task

**DOI:** 10.1121/10.0015356

**Published:** 2022-12-02

**Authors:** Erol J. Ozmeral, Nathan C. Higgins

**Affiliations:** Department of Communication Sciences and Disorders, University of South Florida, Tampa, Florida 33620, USA eozmeral@usf.edu, higgins1@usf.edu

## Abstract

The classic spatial release from masking (SRM) task measures speech recognition thresholds for discrete separation angles between a target and masker. Alternatively, this study used a modified SRM task that adaptively measured the spatial-separation angle needed between a continuous male target stream (speech with digits) and two female masker streams to achieve a specific SRM. On average, 20 young normal-hearing listeners needed less spatial separation for 6 dB release than 9 dB release, and the presence of background babble reduced across-listener variability on the paradigm. Future work is needed to better understand the psychometric properties of this adaptive procedure.

## 1. Introduction

In a complex environment with competing speech, listeners must use varying spectro-temporal and other defining features, like spatial location, to effectively group and segregate auditory objects (Shinn-Cunningham, 2008). An approach to quantifying one's ability to use space to segregate competing auditory streams is to use spatial release from masking (SRM) (Litovsky, 2012). SRM describes the difference in speech reception thresholds (in decibels) for a co-located, compared to a spatially separated, target and masker(s) (either physically, e.g., Litovsky, 2005; or virtually, e.g., Kidd *et al.*, 2010). In the laboratory, listeners can have up to 8 dB or more benefit when separating a masker from a target both in the free field or over headphones (Srinivasan *et al.*, 2020), meaning they can achieve the same speech performance for spatially separated stimuli compared to co-located stimuli even after the target-to-masker ratio (TMR) is reduced by 8 dB. SRM tasks have been widely adopted for assessing binaural function in the laboratory, especially in clinical populations (Arbogast *et al.*, 2005; Gallun *et al.*, 2013), and when virtualized over headphones, they could reasonably be administered in the clinic as well (Jakien *et al.*, 2017; King *et al.*, 2020).

Whereas traditional SRM methods may determine the speech reception advantage for a certain degree of separation between competing speech, they lack precision determining how much spatial separation is needed to sufficiently segregate competing speech. Knowing the required spatial separation to achieve stream segregation, a “functional spatial boundary,” can be used to directly assess underlying spatial-hearing mechanisms and potentially provide meaningful benchmarks for targeted treatment. Previous attempts to measure this boundary have relied on indirectly extracting it from a fitted psychometric curve across multiple spatial separations (e.g., Marrone *et al.*, 2008a). Although that approach can yield spatial separation thresholds for any specific SRM, it can be fundamentally time consuming. The goal of the present study, therefore, was to more quickly and more directly assess the angular separation needed between a target and maskers for a specific SRM.

In a recent study, Ahrens *et al.* (2020) (see Experiment 3) performed an SRM task that adaptively measured speech intelligibility for varying spatial separations between target and masker sentences. This method required experimenters to measure the co-located speech threshold (in dB TMR), and then select a specific achievable benefit (e.g., 6 dB SRM) before adaptively separating the target and maskers to identify the needed spatial separation. Similarly, the present study adopted this more direct approach to measuring a spatial separation threshold, but unlike the method in Ahrens *et al.* (2020) that used sentence materials, the present study used masker and target speech corpora that were presented continuously throughout a run with interleaved digits only in the target stream. The present study also extends the Ahrens *et al.* (2020) method by including a second SRM and conditions with background babble to further our understanding of this task's limits and capabilities as a tool for directly measuring spatial separation thresholds.

Many SRM experiments are conducted using sentence key-word identification as in the coordinate–response–measure (CRM) corpus (Bolia *et al.*, 2000) or with more elaborate matrix–sentence corpora (e.g., Goupell *et al.*, 2018; Ahrens *et al.*, 2020); however, sentence tasks like these are limited to performance assessments on discrete stimulus presentations requiring a distinct, open-ended behavioral response period. Due to the response window break between trials, there is a lack of speech continuity which can limit a listener's ability to sustain an effective auditory stream (Anstis and Saida, 1985; Best *et al.*, 2018). Whereas that stipulation is typically not a major drawback for some research, it can make it less useful for investigations of auditory streaming that are known to break down with large temporal gaps (Bregman *et al.*, 2000). Recently, we developed continuous speech materials for use in dynamic environments amenable to auditory stream segregation paradigms (Ozmeral *et al.*, 2020). In addition to perceptual streaming utility, running speech also avoids pitfalls in hearing aid research experimental paradigms where intermittent speech or noise causes signal processing features to sporadically engage or disengage (Chung, 2004). The present study adopted these continuous speech materials to capitalize on these particular advantages over sentence-based tasks.

## 2. Methods

### 2.1 Participants

Twenty young normal-hearing adults (7 males and 13 females) ages 18.4–26.1 years (mean 22.1, standard deviation 2.0) participated in this study. The listeners all had pure-tone hearing thresholds better than 25 dB HL at octave frequencies up to 8 kHz. The study was approved by the Institutional Review Board of the University of South Florida, and all participants provided written consent and were monetarily compensated.

### 2.2 Apparatus

Listeners were seated on a height-adjustable chair in a large double-walled sound treated room (interior dimensions: 10.0′ × 10.33′ × 6.5′) (Acoustic Systems, Austin, TX) and instructed to always orient their head towards the center speaker. A 15.6″ touchscreen monitor (1080 p) was mounted to the chair arm. Twenty-four KEF (Maidstone, England) Q100 loudspeakers with dual-concentric drivers were evenly arranged in a 360° horizontal array (15° spacing) with a radius of 41″ and an elevation at ear level when the listener was seated in the array center. Outside the room, digital-to-analog conversion was performed by a 24-channel external sound card (MOTU 24ao, Cambridge, MA) routed through three 8-channel amplifiers (Ashly ne8250, Webster, NY). Stimuli were sampled at 44.1 kHz and presented at condition-specific levels as determined in custom matlab (v.2021b; The MathWorks, Inc., Natick, MA) software.

### 2.3 Materials

Speech materials were from the Continuous Number Identification Test (Ozmeral *et al.*, 2020), which consists of roughly 2500 monosyllabic words each recorded from two male talkers and two female talkers. Single-talker speech streams were created by concatenating randomly selected words at a rate of 2 words/s including a 100 ms inter-word-interval. Designated target streams included a numeric target (digits 1 through 10, not including 7) inserted roughly every 3 s. Each of the two masker streams consisted of randomly selected words from the same corpus, excluding all digits. The experimenter instructed participants that the target stream was the male talker, whereas the two female talkers were always the masker streams. The target stream was always located at the front (0° azimuth) while masker streams were limited to a range of 0° to ±90° azimuth. For angles between two fixed loudspeaker positions (15° separation), equal power panning was used to provide better spatial resolution.

### 2.4 Procedure

The study consisted of two primary conditions for determining an individual's spatial boundary: (1) a co-located condition with 1 target (male) and 2 masker (female) streams from the front (0° azimuth), and (2) a spatially separated condition with 1 target from the front and the 2 masker streams split symmetrically to the left and right of center. Unlike prior SRM studies in which speech reception thresholds (in dB TMR) in the spatially separated condition were estimated for a fixed masker azimuth (e.g., Gallun *et al.*, 2013), the present study adaptively changed the azimuth of the maskers for a fixed TMR. For this study, the TMR was individually reduced by 6 dB or 9 dB relative to the threshold estimated in the co-located condition, targeting an SRM of 6 and 9 dB, respectively, and for this reason, the co-located condition was always administered first. In addition to the two primary conditions, a secondary condition also tested listeners with classic spatially separated target and maskers, in which the azimuth of the maskers was fixed at 30° left and right of center. These conditions were included to validate the use of the present stimuli and provide a link to past SRM studies. Listeners performed each condition three times, and the average was recorded as the estimated threshold (in dB TMR for fixed masker-location conditions or angle for the adaptive masker-location condition).

Speech identification performance was assessed using a 2-down, 1-up adaptive procedure that estimated a 70.7% accuracy (Levitt, 1971). In the fixed masker-location conditions, the initial TMR was +12 dB, and initial step size was 2 dB, which was reduced to 1 dB after 3 reversals. In the adaptive masker-location conditions, the initial spatial separation was 45°, and initial step size was 5°, which was reduced to 2° after 3 reversals. Listeners were provided a digital keypad on the touch screen monitor situated above their lap. The numbers 1–10 (but not 7 due to syllable inconsistency with all other numbers), were represented on separate buttons, each roughly 1 square inch. Participants were allowed to self-pace themselves by pressing any button to begin a trial. Trials (each adaptive track) were blocked by spatial condition. Within a trial, listeners were tasked with identifying each digit in the male speech stream coming from the front speaker while ignoring the two female speech streams to the best of their ability. Errant presses of the keypad either before or after a number presentation as well as failure to respond within 2 s of a number presentation resulted in a decrease in the masker levels (fixed masker-location conditions) or a broadening of the masker angles (adaptive masker-location condition). Two sequentially correct responses resulted in an opposite update (increase in masker level or narrowing of masker angle, respectively). A single trial was ended after 12 reversals, and threshold was estimated per trial by the average masker level or angle at the last 9 reversals in the adaptive track. On average, each threshold track was 104 s in duration.

To evaluate the robustness of this SRM task and extend its capabilities, listeners were tested in two different background conditions: (1) quiet background, and (2) multi-talker babble at 55 dB sound pressure level (SPL) (−10 dB SNR). Background stimuli were presented diffusely from four loudspeakers behind the listener at ±165° and ±135°. Listeners completed a single background condition before moving on to another, and the order of background conditions was counterbalanced across participants.

## 3. Results

**Fig. 1.**
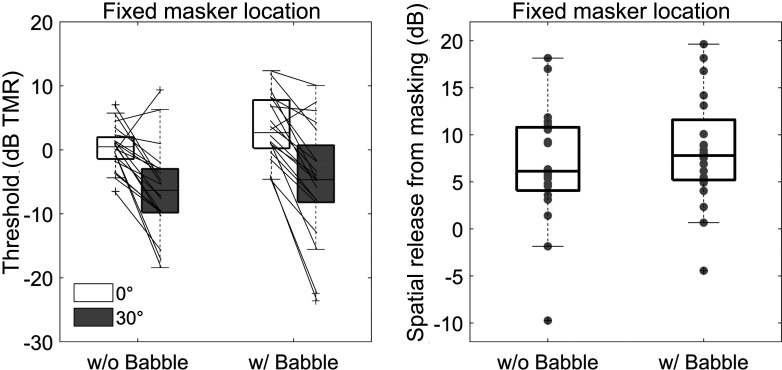
Individual thresholds for a co-located (white) and 30°-separated (gray) fixed masker location. Data in left panel are raw thresholds in dB target-to-masker ratio (TMR); data in right panel are the difference between co-located and spatially separated thresholds (i.e., spatial release from masking [SRM]). Boxplots show median as a horizontal line, whiskers extend from the extreme individual points between 25th and 75th percentile, and crosses indicate outliers (created using boxplot2) (Kearney, 2022).

Figure 1 (left panel) shows the individual and average thresholds for the co-located (0°; white) and fixed spatial separation (30°; gray) conditions with and without background babble present. In the right panel of Fig. 1, SRM is derived as the difference between the co-located and spatially separated conditions in the left panel. As expected, the spatial separation between maskers and target improved listener thresholds. On average, listeners had comparable SRM with (8.3 dB) or without (6.7 dB) babble present. The fixed masker-location thresholds were submitted to a two-way, repeated measures analysis of variance (ANOVA) with two levels of masker angle (0° and 30°) and two levels of background (with and without babble). Results confirmed a main effect of masker location (F[1,19] = 48.7, p < 0.001, η_p_^2^ = 0.72), and there was no effect of the background babble (F[1,19] = 1.9, p = 0.18, η_p_^2^ = 0.09) or any interaction (F[1,19] = 1.0, p = 0.33, η_p_^2^ = 0.05).

**Fig. 2.**
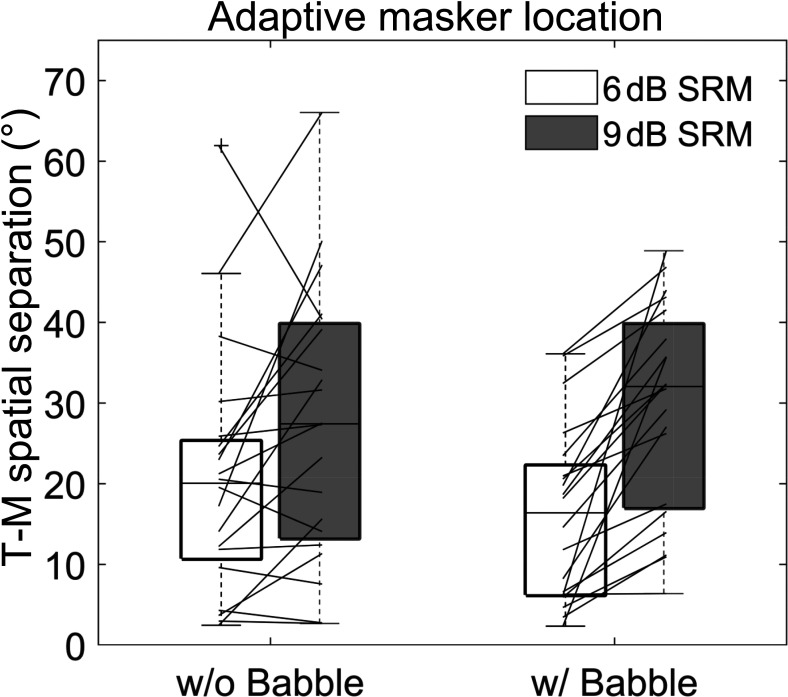
Individual spatial boundary thresholds for 6 dB (white) and 9 dB (gray) release from masking without babble (left) or with babble (right) present. Boxplots show median as a horizontal line, whiskers extend from the extreme individual points between 25th and 75th percentile, and crosses indicate outliers (created using boxplot2) (Kearney, 2022).

Whereas the previous analyses were more analogous to the traditional SRM experiment with fixed masker locations, the data in Fig. 2 represent the individual and average thresholds for the new, adaptive masker-location task. Individual data, like in the fixed masker conditions, had large variability, but there was a clear increase in needed spatial separation for the larger (9 dB; gray) SRM than the smaller (6 dB; white) SRM. On average, without background babble, listeners needed 20.7° to achieve 6 dB SRM, and 27.3° to achieve 9 dB SRM. With babble present, listeners needed 16.1° (6 dB SRM) and 29.5° (9 dB SRM). A two-way, repeated measures ANOVA with two levels of SRM (6 and 9 dB) and two levels of background (with and without babble) reached significance for the main effect of SRM (F[1,19] = 21.4, p < 0.001, η_p_^2^ = 0.53) but not background (F[1,19] = 0.50, p = 0.49, η_p_^2^ = 0.03); and there was a significant interaction between SRM and background (F[1,19] = 6.0, p = 0.024, η_p_^2^ = 0.24). The interaction can be best explained by the smaller threshold difference for the two SRMs when babble was not present compared to when the babble was present.

## 4. Discussion

The present study describes an SRM task that measures the angular separation needed to achieve 6 or 9 dB release between a target stream and competing masker streams. As with classic procedures, this task estimates a listener's threshold for co-located stimuli, and then it directly sets a desired SRM and adaptively tests the angular separation needed to achieve this SRM (similar to Experiment 3 in Ahrens *et al.*, 2020). To get a comparable measure in the classic test, researchers would need to test multiple spatial separations and indirectly extract a desired SRM from a fitted psychometric curve (e.g., Marrone *et al.*, 2008a). Whereas previous SRM tasks have used sentence corpora to probe SRM (e.g., Gallun *et al.*, 2013), including corpora adapted for the clinic (King *et al.*, 2020), the present study used a continuous speech stream that can better target the listener's auditory streaming abilities and could also provide a better tool for assessing adaptive signal processing available in most premium hearing devices.

The effect of spatial separation between a target and maskers (i.e., SRM) can vary widely depending on the utility of spatial cues and the listener's ability to capitalize on those cues (Litovsky, 2012). Spatial cues are particularly useful when a speech target and speech maskers are qualitatively similar, same gender for instance, due to non-energetic factors. In such cases, SRM can be as much as 10 dB or 12 dB at the widest separations for an identification task (Marrone *et al.*, 2008a; Oh *et al.*, 2021). In the present study, maskers were the opposite gender of the target because the running speech materials lacked a comparable voice identifier, like a call sign in the CRM corpus, but there was still between 6.7 and 8.3 dB SRM on average with 30° separation, depending on whether a background babble was present or not. The two maskers were split symmetrically in the frontal hemifield, so it was assumed that monaural head shadow effects were small, but a more systematic investigation of the monaural and binaural effects would be necessary to identify the contributing factors to the observed release (Glyde *et al.*, 2013). Nevertheless, the significant difference between the co-located and separated, fixed masker-location conditions demonstrated the utility of this speech corpus for an SRM task.

In the only other known instance of testing young normal-hearing adults on an SRM task using the separation angle as the tracking variable, Ahrens *et al.* (2020) explored the effect of ambisonic order when virtualizing the spatial location *via* a 24-speaker array. Using 6 dB SRM as the target, listeners averaged between ∼15° and ∼36° spatial separation thresholds depending on how “wide” the target and masker stimuli were perceived. These results are largely consistent with the present study for the 6 dB SRM conditions, and as Ahrens *et al.* (2020) observed, listeners varied widely in their thresholds. One of the hallmarks of speech masking tasks has been considerable individual variability in thresholds (Ruggles *et al.*, 2011; Swaminathan *et al.*, 2015), which may stem from large variability in binaural processing and precision even in normal-hearing listeners (Goupell and Barrett, 2015; Lertpoompunya *et al.*, 2022). In the adaptive masker-location conditions, spatial separation thresholds ranged from 2.4° to 61.9° for 6 dB SRM and 2.7° and 66.0° for 9 dB SRM without babble present. The range was narrower with babble present, but still varied widely across this group of 20 young, normal-hearing listeners. Although the gender of the competing speech was opposite that of the targets, it is possible that non-energetic factors (i.e., information masking) also contributed to the large individual variability (Lutfi *et al.*, 2003). Interestingly, the addition of a background babble had a stabilizing effect across listeners in the present study, such that the group standard deviation reduced from 15.1° to 10.8° in the 6 dB SRM conditions and from 17.0° to 12.9° in the 9 dB SRM conditions. It is possible that the noise reduced audibility of low-level target cues that were used by some but not all listeners to glimpse targets in the two-talker masker, and the presence of the background babble limited this advantage for those listeners (Buss *et al.*, 2017).

The low-level background babble also led to more consistent effects of the targeted SRMs, with all subjects having larger (poorer) spatial separation thresholds in the 9 dB than the 6 dB SRM conditions, which was not always the case without the babble present. It is still unclear why the background babble in particular might have led to poorer thresholds in the 9 dB than the 6 dB SRM conditions, but one possibility is the underlying psychometric curves or position on the curves differ between conditions (with or without babble). This perceptual difference with a low-level, mostly energetic background could be driven by increased effects of informational masking by disrupting listeners' abilities to segregate the target from the maskers (Best *et al.*, 2020). Increased informational masking is another possible reason that greater additional spatial separation is needed with babble present than without it present; however, greater informational masking has been associated with greater across-listener variability which is counter to what we observed. The source of this contradiction remains unsolved.

Last, although the test is relatively quick (1.7 min per adaptive track) and provides insight into what we have called a functional spatial boundary, in its current form, it is not without its limitations. Due to the constraints on hardware and space availability, the task is not very practical for use in the clinic despite its potential importance in comparing effects of devices. Another limitation of the task is that experimenters must choose an SRM that is within the listener's capabilities for the available spatial separations. For example, in the fixed masker-location conditions, it was clear that some subjects failed to receive much benefit from the 30° separation, and although all subjects converged on a threshold in the adaptive masker-location conditions, it is conceivable that listeners in special populations (e.g., older, hearing-impaired, cochlear-implant users, etc.) might not receive as much as 6 dB SRM in any situation (Marrone *et al.*, 2008b). Finally, the underlying psychometric properties of the current SRM task are still unknown, and full knowledge of that psychometric curve might inform future versions of the experiment to optimize sensitivity. Ahrens *et al.* (2020) used a non-linear function and slope parameter to adjust the step sizes as recommended by Brand and Kollmeier (2002), whereas this study used of consistent 5° and 2° step sizes. Without having the underlying psychometric functions of the fixed TMR conditions, it is not possible to estimate the perceived magnitude change relative to the fixed location conditions that used 2 dB and 1 dB step sizes.

To conclude, we conducted a test that directly measures the spatial separation needed for a specific spatial release from masking when using a continuous target and two opposite-gender masker streams. This test measures an individuals' co-located threshold in the same fashion as a classic SRM procedure, then reduces the target-to-masker ratio and adaptively changes the target-to-masker spatial separation to achieve the same speech recognition performance. We used continuous speech stimuli to investigate auditory streaming, and results were consistent with past methods using sentence key-word identification. Knowing the spatial separation needed to achieve a given SRM will be useful in diagnoses and treatment based on individual differences. This paradigm also has the potential to provide critical information to researchers when testing various device programs by directly assessing changes, if any, to the spatial boundary, without testing at different stimulus levels.

## References

[1] Ahrens, A. , Marschall, M. , and Dau, T. (2020). “ The effect of spatial energy spread on sound image size and speech intelligibility,” J. Acoust. Soc. Am. 147, 1368–1378. 10.1121/10.0000747 32237851

[2] Anstis, S. , and Saida, S. (1985). “ Adaptation to auditory streaming of frequency-modulated tones,” J. Exp. Psychol. Hum. Percept. Perform. 11, 257–271. 10.1037/0096-1523.11.3.257

[3] Arbogast, T. L. , Mason, C. R. , and Kidd, G., Jr. (2005). “ The effect of spatial separation on informational masking of speech in normal-hearing and hearing-impaired listeners,” J. Acoust. Soc. Am. 117, 2169–2180. 10.1121/1.1861598 15898658

[4] Best, V. , Conroy, C. , and Kidd, G. (2020). “ Can background noise increase the informational masking in a speech mixture?,” J. Acoust. Soc. Am. 147, EL144–EL150. 10.1121/10.0000719 32113285 PMC7015733

[5] Best, V. , Swaminathan, J. , Kopčo, N. , Roverud, E. , and Shinn-Cunningham, B. (2018). “ A ‘buildup’ of speech intelligibility in listeners with normal hearing and hearing loss,” Trends Hear. 22, 233121651880751. 10.1177/2331216518807519 PMC6201174 30353783

[6] Bolia, R. , Nelson, W. , Ericson, M. , and Simpson, B. (2000). “ A speech corpus for multitalker communications research,” J. Acoust. Soc. Am. 107, 1065–1066. 10.1121/1.428288 10687719

[7] Brand, T. , and Kollmeier, B. (2002). “ Efficient adaptive procedures for threshold and concurrent slope estimates for psychophysics and speech intelligibility tests,” J. Acoust. Soc. Am. 111, 2801–2810. 10.1121/1.1479152 12083215

[8] Bregman, A. S. , Ahad, P. A. , Crum, P. A. C. , and O'Reilly, J. (2000). “ Effects of time intervals and tone durations on auditory stream segregation,” Percept. Psychophys. 62, 626–636. 10.3758/BF03212114 10909253

[9] Buss, E. , Leibold, L. J. , Porter, H. L. , and Grose, J. H. (2017). “ Speech recognition in one- and two-talker maskers in school-age children and adults: Development of perceptual masking and glimpsing,” J. Acoust. Soc. Am. 141, 2650–2660. 10.1121/1.4979936 28464682 PMC5391283

[10] Chung, K. (2004). “ Challenges and recent developments in hearing aids. Part I. Speech understanding in noise, microphone technologies and noise reduction algorithms,” Trends Amplif. 8, 83–124. 10.1177/108471380400800302 15678225 PMC4111442

[11] Gallun, F. J. , Diedesch, A. C. , Kampel, S. D. , and Jakien, K. M. (2013). “ Independent impacts of age and hearing loss on spatial release in a complex auditory environment,” Front Neurosci. 7, 252. 10.3389/fnins.2013.00252 24391535 PMC3870327

[12] Glyde, H. , Buchholz, J. , Dillon, H. , Best, V. , Hickson, L. , and Cameron, S. (2013). “ The effect of better-ear glimpsing on spatial release from masking,” J. Acoust. Soc. Am. 134, 2937–2945. 10.1121/1.4817930 24116429

[13] Goupell, M. J. , and Barrett, M. E. (2015). “ Untrained listeners experience difficulty detecting interaural correlation changes in narrowband noises,” J. Acoust. Soc. Am. 138, EL120–EL125. 10.1121/1.4923014 26233053 PMC4514722

[14] Goupell, M. J. , Stoelb, C. A. , Kan, A. , and Litovsky, R. Y. (2018). “ The effect of simulated interaural frequency mismatch on speech understanding and spatial release from masking,” Ear Hear. 39, 895–905. 10.1097/AUD.0000000000000541 29337763 PMC6046281

[15] Jakien, K. M. , Kampel, S. D. , Stansell, M. M. , and Gallun, F. J. (2017). “ Validating a rapid, automated test of spatial release from masking,” Am. J. Audiol. 26, 507–518. 10.1044/2017_AJA-17-0013 28973106 PMC5968328

[16] Kearney, K. (2022). “ boxplot2,” https://github.com/kakearney/boxplot2-pkg (Last viewed July 7, 2022).

[17] Kidd, G., Jr. , Mason, C. R. , Best, V. , and Marrone, N. (2010). “ Stimulus factors influencing spatial release from speech-on-speech masking,” J. Acoust. Soc. Am. 128, 1965–1978. 10.1121/1.3478781 20968368 PMC2981113

[18] King, G. , Corbin, N. E. , Leibold, L. J. , and Buss, E. (2020). “ Spatial release from masking using clinical corpora: Sentence recognition in a colocated or spatially separated speech masker,” J. Am. Acad. Audiol. 31, 271–276. 10.3766/jaaa.19018 31589139 PMC7117988

[19] Lertpoompunya, A. , Ozmeral, E. J. , Higgins, N. C. , Eddins, A. C. , and Eddins, D. A. (2022). “ Large group differences in binaural sensitivity are represented in preattentive responses from auditory cortex,” J. Neurophysiol. 127, 660–672. 10.1152/jn.00360.2021 35108112 PMC8896993

[20] Levitt, H. (1971). “ Transformed up-down methods in psychoacoustics,” J. Acoust. Soc. Am. 49, 467–477. 10.1121/1.1912375 5541744

[21] Litovsky, R. Y. (2005). “ Speech intelligibility and spatial release from masking in young children,” J. Acoust. Soc. Am. 117, 3091–3099. 10.1121/1.1873913 15957777

[22] Litovsky, R. Y. (2012). “ Spatial release from masking,” Acoust. Today 8, 18–25. 10.1121/1.4729575

[23] Lutfi, R. A. , Kistler, D. J. , Oh, E. L. , Wightman, F. L. , and Callahan, M. R. (2003). “ One factor underlies individual differences in auditory informational masking within and across age groups,” Percept. Psychophys. 65, 396–406. 10.3758/BF03194571 12785070 PMC2819167

[24] Marrone, N. , Mason, C. R. , and Kidd, G., Jr. (2008a). “ Tuning in the spatial dimension: Evidence from a masked speech identification task,” J. Acoust. Soc. Am. 124, 1146–1158. 10.1121/1.2945710 18681603 PMC2809679

[25] Marrone, N. , Mason, C. R. , and Kidd, G., Jr. (2008b). “ The effects of hearing loss and age on the benefit of spatial separation between multiple talkers in reverberant rooms,” J. Acoust. Soc. Am. 124, 3064–3075. 10.1121/1.2980441 19045792 PMC2736722

[26] Oh, Y. , Bridges, S. E. , Schoenfeld, H. , Layne, A. O. , and Eddins, D. (2021). “ Interaction between voice-gender difference and spatial separation in release from masking in multi-talker listening environments,” JASA Express Lett. 1, 084404. 10.1121/10.0005831 34713273 PMC8547139

[27] Ozmeral, E. J. , Hoover, E. C. , Gabbidon, P. , and Eddins, D. A. (2020). “ Development of the Continuous Number Identification Test (CNIT): Feasibility of dynamic assessment of speech intelligibility,” Int. J. Audiol. 59, 434–442. 10.1080/14992027.2020.1718782 32003257

[28] Ruggles, D. , Bharadwaj, H. , and Shinn-Cunningham, B. G. (2011). “ Normal hearing is not enough to guarantee robust encoding of suprathreshold features important in everyday communication,” Proc. Natl. Acad. Sci. U.S.A. 108, 15516–15521. 10.1073/pnas.1108912108 21844339 PMC3174666

[29] Shinn-Cunningham, B. G. (2008). “ Object-based auditory and visual attention,” Trends Cogn. Sci. 12, 182–186. 10.1016/j.tics.2008.02.003 18396091 PMC2699558

[30] Srinivasan, N. K. , Holtz, A. , and Gallun, F. J. (2020). “ Comparing spatial release from masking using traditional methods and portable automated rapid testing iPad app,” Am. J. Audiol. 29, 907–915. 10.1044/2020_AJA-20-00078 33197327 PMC8608168

[31] Swaminathan, J. , Mason, C. R. , Streeter, T. M. , Best, V. , Kidd, G., Jr. , and Patel, A. D. (2015). “ Musical training, individual differences and the cocktail party problem,” Sci. Rep. 5, 11628. 10.1038/srep11628 26112910 PMC4481518

